# Antibacterial Activity of Shikimic Acid from Pine Needles of *Cedrus deodara* against *Staphylococcus aureus* through Damage to Cell Membrane

**DOI:** 10.3390/ijms161126015

**Published:** 2015-11-13

**Authors:** Jinrong Bai, Yanping Wu, Xiaoyan Liu, Kai Zhong, Yina Huang, Hong Gao

**Affiliations:** 1 Department of Food Science and Technology, College of Light Industry, Textile and Food Engineering, Sichuan University, Chengdu 610065, China; baijinrong01@hotmail.com (J.B.); wyp9202@163.com (Y.W.); lxy19831231@163.com (X.L.); 2 Department of Public Health, West China Medical School, Sichuan University, Chengdu 610041, China; dir0932@sina.com

**Keywords:** shikimic acid, antibacterial activity, *Staphylococcus aureus*, membrane damage, membrane proteins, membrane lipids

## Abstract

Shikimic acid (SA) has been reported to possess antibacterial activity against *Staphylococcus aureus*, whereas the mode of action of SA is still elusive. In this study, the antibacterial activity and mechanism of SA toward *S. aureus* by cell membrane damage was investigated. After SA treatment, massive K^+^ and nucleotide leakage from *S. aureus*, and a significant change in the membrane potential was observed, suggesting SA may act on the membrane by destroying the cell membrane permeability. Through transmission electron microscopic observations we further confirmed that SA can disrupt the cell membrane and membrane integrity. Meanwhile, SA was found to be capable of reducing the membrane fluidity of the *S. aureus* cell. Moreover, the fluorescence experiments indicated that SA could quench fluorescence of Phe residues of the membrane proteins, thus demonstrating that SA can bind to *S. aureus* membrane proteins. Therefore, these results showed the antibacterial activity of SA against *S. aureus* could be caused by the interactions of SA with *S. aureus* membrane proteins and lipids, resulting in causing cell membrane dysfunction and bacterial damage or even death. This study reveals the potential use of SA as an antibacterial agent.

## 1. Introduction

Severe foodborne disease and intoxications are a widespread and growing public health problem [1]. Nowadays, this problem has caused more attentions of governments and food industry than a few decades ago. Foodborne disease is one of the major causes of illness and death, and about 48 million cases of foodborne diseases are estimated to occur each year in the United States [2,3]. Among them, staphylococcal foodborne disease is a common and major foodborne disease worldwide [4]. *Staphylococcus aureus* is a significant pathogen for foodborne disease, and caused many people illness and death every year [3]. *S. aureus* can cause a series of illnesses from mild skin infections to more severe life-threatening diseases, such as osteomyelitis, pneumonia, and septicemia [5]. One of the most common toxins in foodborne intoxication is staphylococcal enterotoxins, which can cause intense diarrhea, nausea, vomiting, and abdominal pain [6]. Therefore, the control of this pathogen is a major concern for people and food industry.

Some conventional physical treatments, including thermal inactivation, irradiation, chained cold treatment, and high-sugar or -salt treatments, have been applied to control *S. aureus*. However, some physical treatments may cause adverse effects on the food such as color change, browning, reductions of aroma nutrients and functional substances, and others [7]. In addition to physical treatments, chemical preservatives are also used in the food industry to control foodborne pathogens and to extend food shelf-lives [8]. While chemical preservatives are convenient for use, their safety and potential health issues have been a controversial topic. Further, indiscriminate use of antibiotics contributed to inducing antibiotic-resistant pathogens. Therefore, there is an enhanced tendency to use safe and efficient natural antimicrobials for the control of foodborne pathogens.

Shikimic acid (SA) is an important organic acid not only as a key intermediate in the biosynthesis of aromatic compounds, but also as a starting material in the chemical synthesis of useful compounds. With the sudden outbreak of flu, SA has aroused unprecedented attention as a key material in the synthesis of antiviral drug Tamiflu^@^, which is effective in antiviral infections such as bird flu and swine flu [9]. The occurrence of SA spreads widely in plants, and extremely abundant in Chinese star anise [10]. In the biological studies, SA has been reported to have anti-inflammatory, analgesia, antioxidant, antithrombotic, and antibacterial activities [11,12,13,14]. In a previous study, we found SA as a major antibacterial compound against *S. aureus* in the water extract obtained from pine needles of *Cedrus deodara* [15].

So far, SA has been extensively studied and applied, but few literatures have reported its antibacterial mechanism against *S. aureus* specifically. In order to fully understand the mode of action and for the further application of SA, the antibacterial mechanism of SA against *S. aureus* was explored. Importantly, it is the first time to elucidate the mode of action of SA against *S. aureus* by investigating the cell membrane permeability, membrane potential, membrane integrity, cell morphology, membrane fluidity, and membrane protein.

## 2. Results and Discussion

### 2.1. Antibacterial Activity of Shikimic Acid (SA) to Staphylococcus aureus (S. aureus)

Minimal inhibition concentration (MIC) of SA against *S. aureus* American type culture collection (ATCC) 6538 was determined to be 2.5 mg/mL.

### 2.2. Leakage of Cell Constituents Induced by SA

**Figure 1:**
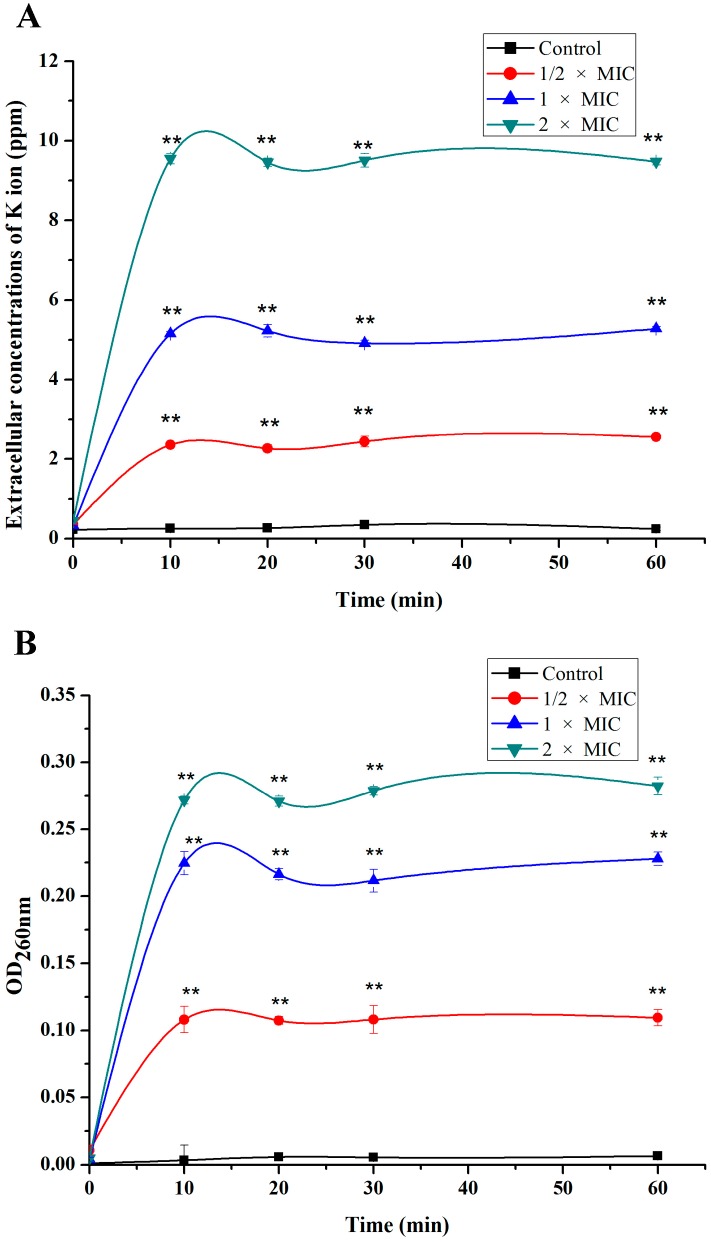
Effect of shikimic acid on the amounts of K^+^ (**A**) and the cell nucleotide (**B**) leakage from *S. aureus* American type culture collection (ATCC) 6538. Data are expressed as mean ± standard deviation. ** *p* < 0.01, compared with control. Minimal inhibition concentration (MIC) is defined to be the lowest concentration of shikimic acid (SA) capable of inhibiting the tested bacterial visible growth.

To demonstrate the effect of SA on the membrane permeability, the amount of K^+^ efflux and nucleotide leakage from *S. aureus* were measured. After SA treatment, the release of K^+^ and nucleotide from *S. aureus* cells increased immediately and significantly, and K^+^ efflux and nucleotide leakage almost remained constant after 10 min of incubation (Figure 1). Moreover, with the increase of SA concentration, the amounts of K^+^ efflux and nucleotide leakage increased correspondingly. Accordingly, SA induced a rapid K^+^ and nucleotide release from *S. aureus*, reaching the maximal efflux after only 10 min treatment. The result shows that SA probably has an ability to influence the membrane permeability. When membrane permeability changes and membrane becomes damaged, some ions, nucleotides, and proteins could leak out. Hence, SA may have destroyed the *S. aureus* K^+^ channels, leading to the massive effluxes of K^+^ from *S. aureus.* Additionally, SA rapidly increased membrane permeability and damaged the membrane, which revealed that SA possessed a rapid and direct antibacterial effect against *S. aureus*. Many other antibacterial agents exhibited similar actions on different bacteria. It is reported that peptides from barbel protein hydrolysates show the ability to disturb the cell membrane and to cause releasing K^+^ extracellularly [16]. Chlorogenic acid was reported to induce an immediate and massive efflux of K^+^ and nucleotides from *S. dysenteriae* and *S. pneumoniae* by increasing plasma membrane permeabilization [17]. These findings support our results and speculation that SA probably acts on the membrane and increases the permeability of cell membranes.

### 2.3. Effect of SA on Membrane Potential

**Figure 2:**
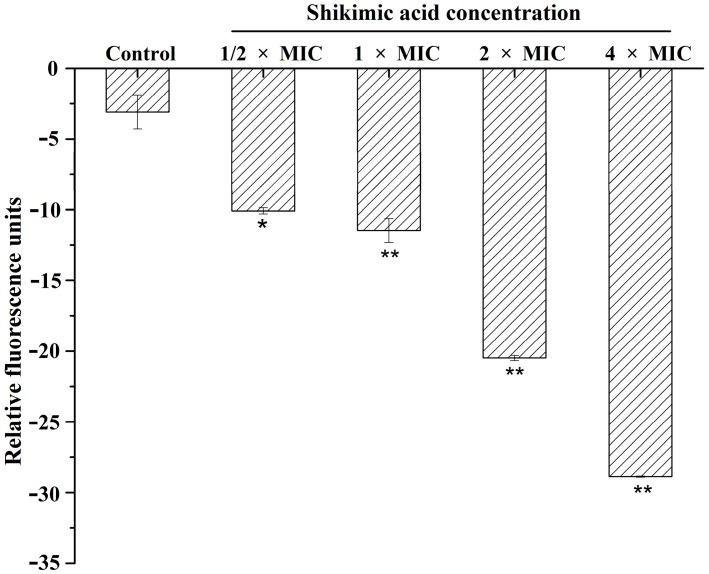
Effect of shikimic acid on the membrane potential of *S. aureus* ATCC 6538. Data are expressed as mean ± standard deviation. * *p* < 0.05, ** *p* < 0.01, compared with control.

The changes of membrane potential were monitored to study the mode of action of SA. DiBAC4(3), a kind of fluorescent probe, is used for monitoring the changes of membrane potential. When membrane becomes depolarized, the dye crosses the cell membrane and fluorescence enhances. Conversely, fluorescence decrease in the state of membrane hyperpolarization [18]. As shown in Figure 2, fluorescence decreased when *S. aureus* cells were treated with SA in a dose-dependent manner. So, the result implied that SA hyperpolarized cell membrane of *S. aureus* and changed the cell membrane permeability. A previous study has indicated that chlorogenic acid may affect the *S. aureus* cell membrane structure and cause the cell membrane hyperpolarization [19]. It is also reported that chlorogenic acid has been reported to disrupt the cell membrane and then depolarize the bacterial cell membrane of *S. pneumonia* and *S. dysenteriae* [20]. Although some discrepancy is noticed between the previous two reports describing the effects of chlorogenic acid on cell membrane polarization, antibacterial agents showed effects on cell membrane by changing its polarization. Combining the above results on K^+^ efflux with our observation that K^+^ was released from *S. aureus* cell by SA in a dose-responding manner, we suggest that SA induces the hyperpolarization of cell membrane. Hence, it was suggested that SA may have an ability to change the membrane potential of *S. aureus*.

### 2.4. Membrane Integrity

To further characterize the antibacterial action of SA on the *S. aureus* membrane integrity, flow cytometric analysis was performed with LIVE/DEAD BacLight bacterial viability kit. The kit is composed of two kinds of nucleic acid dyes: green fluorescent SYTO 9 is able to penetrate all bacterial membrane, red fluorescent propidium iodide (PI) only enters into cells with damaged membrane [21]. Due to the strong fluorescence signal of SYTO 9 in 600 nm except for 525 nm, it overlaps with the fluorescence signal of PI in 620 nm. Therefore, the cloud of untreated samples of *S. aureus* is in the upper right quadrant. [22]. Additionally, when the cell membrane is damaged, PI enters into the cell and produces red fluorescence, which caused intensity of SYTO 9 fluorescence to become lower. Due to fluorescence resonance energy transfer, the intensity of red fluorescence becomes lower, since green fluorescence is in lower intensity [23]. To analyze cell membrane integrity, the flow cytometry analyses were performed in two regions: R1 (membrane damaged and dead cells region), and R2 (viable cells region).

**Figure 3:**
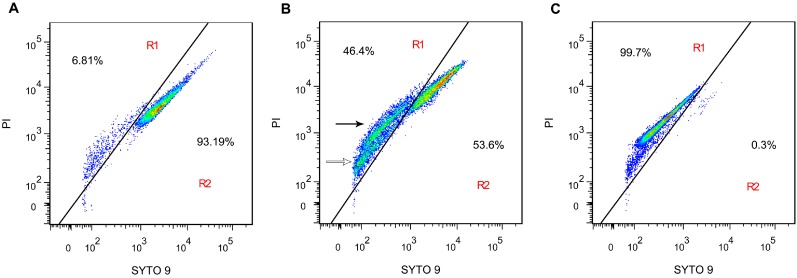
Flow cytometric analysis of *S. aureus* ATCC 6538 stained by SYTO 9 and propidium iodide (PI). (**A**) untreated; (**B**) treated with shikimic acid at 1 × MIC for 3 h; and (**C**) positive control, treated with 70% isopropanol for 3 h. R1, membrane disrupted cells gate; R2, viable cells gate. Two fluorescent populations are observed in R1 with a higher fluorescent intensity (black arrow) and lower fluorescent intensity (white arrow). The red, green, and blue colors represent the decrement of cell density.

Following 3 h incubation with 1 × MIC of SA, the population of cells with intact membrane was 53.6% (Figure 3B), whereas that was 93.19% for the untreated sample (Figure 3A). The population of cells with damaged membrane increased 6.81% in the untreated sample to 46.4% with 1 × MIC of SA treatment, indicating that cell membrane integrity was lost after exposure to SA. Significantly, there were two fluorescent populations in R1 region. One population (Figure 3B, black arrow) had higher intensity in both red and green fluorescence than the other population had (Figure 3B, white arrow). This pattern likely occurs because the cells were stained by different concentrations of SYTO 9 and PI, resulting from the short of nucleic acids in the second population cells [23]. Overall, the above results further revealed that the cell membrane was the target of SA in its antibacterial action.

### 2.5. Transmission Electron Microscopy (TEM)

**Figure 4:**
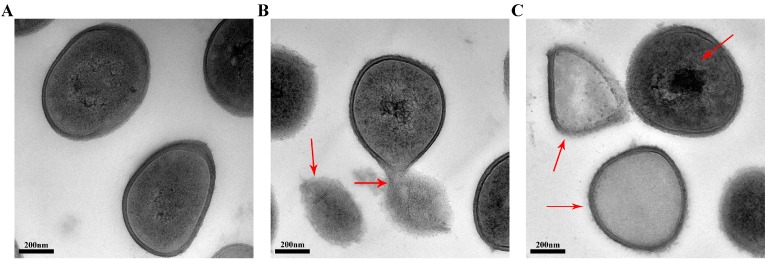
Transmission electron micrographs of *S. aureus* ATCC 6538 cells. (**A**) untreated bacteria; and (**B**,**C**) bacteria treated with shikimic acid at 1 × MIC for 12 h.

To visually disclose the action of SA on cell membrane, the effect of SA on *S. aureus* morphology and the membrane was observed by TEM. Compared with untreated *S. aureus* cells, the cellular morphology of *S. aureus* treated with SA changed significantly. The untreated *S. aureus* cells maintained plump, globose, and integrity, and the *S. aureus* wall and membrane were intact, appearing as well-defined cell membranes. Moreover, normal cells showed homogeneous electron density (Figure 4A). In contrast, the *S. aureus* cells treated with SA showed distorted shape and lost the clear boundary of cell membrane. The cell membrane permeability changed, or may even been damaged, leading to the leakage of cell contents into the media, resulting in electron translucence of the cell (Figure 4B). It was easy to find cell debris and electron-dense particles around the disrupted cells, which was likely due to the damaged and lysed bacteria. Additionally, probably because of cytoplasmic disruption, the cells treated with SA showed electron density heterogeneity (Figure 4C). Cell walls and membranes can maintain cell morphology and control normal cellular functions. Through TEM observations of the *S. aureus* cells, we further verified that SA can disrupt the cell membrane, including cell permeability and cell integrity, which probably causes cell growth inhibition and death.

### 2.6. Effect of SA on the S. aureus Cell Membrane Fluidity

**Figure 5:**
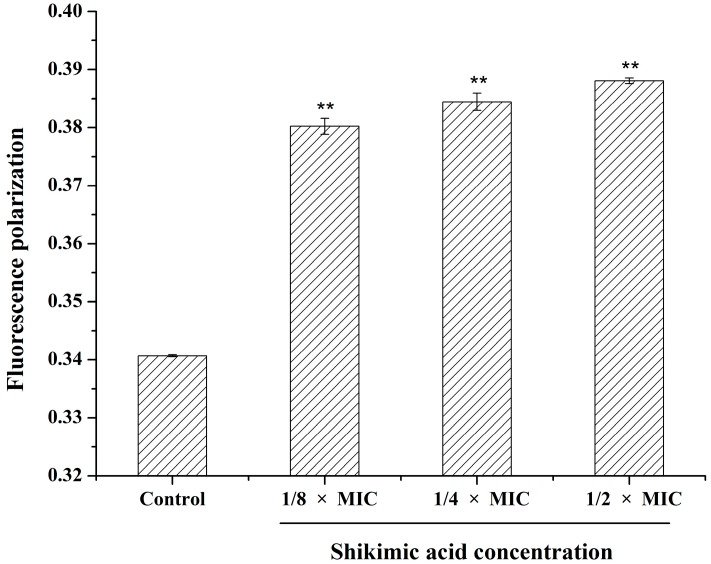
Effect of shikimic acid on the fluidity of membrane lipid of *S. aureus* ATCC 6538. Data are expressed as mean ± standard deviation. ** *p* < 0.01, compared with control.

To further assess the mechanism of SA on the *S. aureus* membrane, we measured the fluorescence polarization of DPH to test the cells’ membrane fluidity. Polarization values could reflect the changes in membrane fluidity. The DPH is a kind of hydrophobic and non-fluorescent probe, which can bind to the cell membrane lipophilic tails and emit strong fluorescence when the cell membrane is intact [24,25]. The higher fluorescence polarization indicates the lower membrane fluidity. As shown in Figure 5, fluorescence polarization values increased with the concentration of SA from 1/8 × MIC to 1/2 × MIC, suggesting a reduction of membrane fluidity. So, SA was capable of affecting the fluidity of hydrophobic membrane interface of the *S. aureus* cell. Membrane fluidity has many important functions, such as transferring signals, energy transformation, and nutrient transportation, which are closely related to membrane fluidity. Hence, membrane fluidity stability performs a significant role to maintain the normal function of cell [26]. The above results indicated that SA may damage the structure of cell membrane lipid bilayers and hinder the cell modulation of the membrane composition, resulting in a decrease of membrane fluidity, increase of cell permeability, and partial solubility of the membrane.

### 2.7. Effect of SA on the S. aureus Cell Membrane Protein

**Figure 6:**
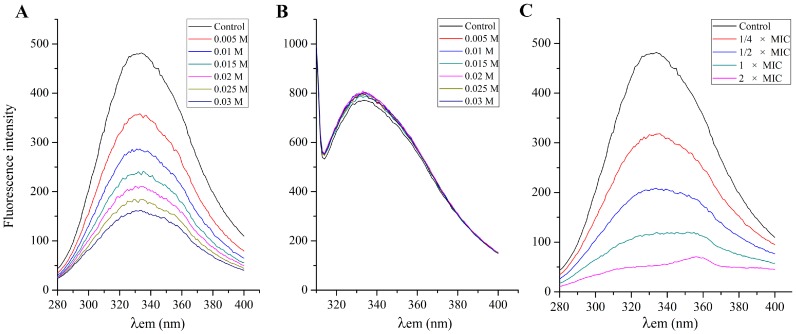
Effect of KI on fluorescence intensity of the *S. aureus* ATCC 6538 membrane protein at λ_ex_ 258 nm (**A**) and λ_ex_ 296 nm (**B**); effect of shikimic acid on fluorescence intensity of the *S. aureus* ATCC 6538 membrane protein at λ_ex_ 258 nm (**C**). Concentrations of KI (from the top down) were 0.005, 0.01, 0.015, 0.02, 0.025 and 0.03 M, respectively. Concentrations of shikimic acid (from the top down) were 1/4 × MIC, 1/2 × MIC, 1 × MIC and 2 × MIC, respectively.

Cell membrane exhibits fluorescence due to tryptophan (Trp), tyrosine (Tyr) and phenylalanine (Phe) residues. Some amino acid residues are situated inside or in the crevice of cell membrane proteins, while some amino acid residues are situated on the cell membrane protein surface [27]. KI is widely used to examine the location of amino acid residues. I^−^ can directly bind to the amino acid residues that locate on the surface of cell membrane protein, and emission fluorescence will be decreased and quenched. On the other hand, I^−^ cannot directly bind to the amino acid residues that are located inside or in the crevice of cell membrane proteins, and fluorescence spectra will not be affected [27]. Figure 6A,B showed the effect of KI on fluorescence intensity of Tyr and Phe residues at λ_ex_ 258 nm and λ_ex_ 296 nm, respectively. Fluorescence intensity of Phe residues decreased dramatically because I^−^ could bind to Phe residues partly, which suggested that some of the Phe residues situated on the *S. aureus* cell membrane surface. However, with the increased of concentration of KI, fluorescence spectra of Tyr residues had no change, which suggested that Tyr residues primarily situated *S. aureus* cell membrane inside or in the crevice. Since the excitation peak of Trp residues is close to the excitation peak of Tyr, the effect of quenching agent on the fluorescence of Trp was similar to that of Tyr. Therefore, the change of Phe residue fluorescence intensity was detected to test whether SA interacts with the cell membrane protein or not.

When *S. aureus* cells are exposed to antibacterial agents, the cell membrane protein conformation changed, and the emission fluorescence of Phe residues would be decreased and quenched. Moreover, the Phe residues would be exposed from inside of membrane proteins, which led to the changes of emission fluorescence intensity and fluorescence spectra. Therefore, fluorescence intensity of Phe residues was selected to reflect whether SA interacts with cell membrane protein [28]. With the concentration of SA ranging from 1/4 × MIC to 2 × MIC, the Phe residues emission fluorescence intensity decreased correspondingly, and even close to zero (Figure 6C). The result demonstrated that SA may make the Phe residues situated in the crevice or inside of the cell membrane protein exposed outside of the membrane protein. In addition to the decrement of fluorescence intensity, when the concentration of SA was set at 1 × MIC or 2 × MIC, the emission fluorescence spectra of Phe residues significantly changed as compared to the control. The above results indicated that SA could quench fluorescence of Phe residues and change the conformation and structure of cell membrane protein. In this regard, the antibacterial action of SA may be mainly due to destruction of the cell membrane proteins, which influenced the normal function of the cell membrane and resulted in bacterial damage or even death. Hence, it was implied that membrane proteins would be the target molecules on the cell surface for the action of SA against of *S.* *aureus*.

## 3. Materials and Methods

### 3.1. Materials

SA was obtained from *C. deodara* pine needles according to the previous report [15], and prepared in solutions with distilled water for use in all experiments. All other chemicals were of analytical grade.

### 3.2. Assay for Antibacterial Activity

The strain *S. aureus* ATCC 6538 was purchased from the China Medical Culture Collection Center (Beijing, China). *S. aureus* was inoculated in nutrition broth (NB) containing 1% tryptone, 0.3% beef extract, and 0.5% NaCl. The antibacterial activity assay was performed using the method of microdilution broth with minor modifications [19]. Briefly, logarithmic phase *S. aureus* cells were diluted with fresh medium to a density of 1 × 10^6^ CFU/mL. Bacterial suspensions (100 μL) and of SA (100 μL) were gently added into the 96-well plates, then the bacterial suspensions were incubated at 37 °C for 12 h. The final concentration of SA was in range from 0.625 to 10 mg/mL. Minimal inhibition concentration (MIC) is defined to be the lowest concentration of SA capable of inhibiting the tested bacterial visible growth.

### 3.3. Measurement of the Potassium Efflux

The assay of the potassium efflux was performed following the reported procedure [29] with a few modifications. In brief, the logarithmic phase *S. aureus* cells was collected by centrifugation at 5000× *g* for 5 min at 4 °C, washed twice, and re-suspended to make 1 × 10^6^ CFU/mL in 0.85% saline solution. Then, 20 mL of the suspensions were incubated with three concentrations of SA (1/2 × MIC, 1 × MIC, 2 × MIC) at 37 °C for different times. Sampling was performed at 0, 10, 20, 30, and 60 min after the start of incubation, and the solutions were filtered through a 0.22 μm filter membrane. The concentration of released K^+^ was measured using an atomic absorption spectrometer (PinAAcle 900, PerkinElmer, Waltham, MA, USA).

### 3.4. Measurement of the Nucleotide Leakage

The nucleotide leakage from *S. aureus* was determined following the reported method [30] with a slight modification. Logarithmic phase *S. aureus* cells were collected by centrifugation at 5000× *g* for 5 min at 4 °C, washed twice, and re-suspended to make 1 × 10^6^ CFU/mL in 0.85% saline solution. Then, 20 mL of the suspensions were incubated with three concentrations of SA (1/2 × MIC, 1 × MIC, 2 × MIC) at 37 °C for different time. Sampling was performed at 0, 10, 20, 30, and 60 min after the start of incubation, and the solutions were filtered through a 0.22 μm filter membrane. The filtrates were measured by a microplate reader (SpectraMax 190, Molecular Devices, Sunnyvale, CA, USA) at 260 nm.

### 3.5. Membrane Potential Assay

The membrane potential assay was carried out using the reported method with a few modifications [31]. Briefly, Logarithmic phase *S. aureus* cells were diluted with fresh medium to a density of 1 × 10^7^ CFU/mL. Three milliliters of suspensions were incubated with four different concentrations of SA (1/2 × MIC, 1 × MIC, 2 × MIC, 4 × MIC) at room temperature for 10 min. An amount of 0.5 μg/mL of *bis*-(1,3-dibutylbarbituric acid) trimethine oxonol (DiBAC4(3); Life Technologies, Eugene, OR, USA), was then added and incubated for 5 min. The fluorescence was measured using a fluorescence spectrophotometer (G9800A, Agilent, Santa Clara, CA, USA). The excitation was set at 492 nm and emission was set at 515 nm.

### 3.6. Membrane Integrity

The effect of SA on *S. aureus* membrane integrity was determined using flow cytometry according to the reported method with slight modifications [21]. The LIVE/DEAD BacLight bacterial viability kit (Life Technologies, Eugene, OR, USA) contains two stains, component A (SYTO 9 dye, 3.34 mM) and component B (Propidium iodide, 20 mM). Equal volumes of two stains were mixed and stored at −25 °C until use. Logarithmic phase *S. aureus* cells were washed twice, and re-suspended at a final density of 1 × 10^9^ CFU/mL in 0.85% saline solution. Then, 20 mL of suspensions were incubated with 1 × MIC of SA at 37 °C. After three hours, the cells were washed twice and re-suspended at a final density of 1 × 10^6^ CFU/mL in 0.85% saline solution. An amount of 1 mL of cell suspension was stained with 3 μL of the dye mixture. Subsequently, the samples were incubated in the dark for 15 min at room temperature. The proportions of live and death cells were analyzed by a flow cytometer (FACSVerse, Becton Dickinson, Franklin Lakes, NJ, USA). The green fluorescent SYTO 9 were examined using a 525 nm channel, and the red fluorescent PI were examined using a 620 nm channel. The experiment collected 50,000 cell events with a low flow rate. The suspensions without SA treatment were used as the negative control, and the suspensions treated with 70% isopropanol were used as the positive control.

### 3.7. TEM

Logarithmic phase *S. aureus* cells were treated with SA at 1 × MIC at 37 °C for 12 h. The bacterial pellets were then treated as previously reported [15]. The thin sections were observed by TEM (H-600IV, Hitachi, Tokyo , Japan).

### 3.8. Membrane Fluidity Assay

Membrane fluidity can be examined by detecting fluorescence polarization of 1,6-diphenyl-1,3,5-hexatriene (DPH; Sigma-Aldrich, St. Louis, MO, USA). The assay was carried out as previously reported with minor modifications [27]. Briefly, logarithmic phase *S. aureus* cells were washed twice and re-suspended at a final density of 1 × 10^9^ CFU/mL in 0.85% saline solution. Cell suspensions were incubated with three different concentrations of SA (1/8 × MIC, 1/4 × MIC, 1/2 × MIC) at room temperature for 1 h. After DPH was added to the above solution to reach a final concentration of 10^−6^ M, the suspensions were incubated for 30 min at room temperature again. Fluorescence polarization was measured by a microplate reader (Synergy H1, BioTek, Winooski, VT, USA). The fluorescence excitation and fluorescence emission were 360 ± 40 nm and 460 ± 40 nm. The fluorescence polarization (*p*) value is calculated by the following formula [32]:

(1) *p* = (*I*_VV_ – *GI*_VH_)/(*I*_VV_ + *GI*_VH_)

where *I*_VV_ is the vertical excitation fluorescence intensity and *I*_VH_ is the horizontal excitation fluorescence intensity, when the fluorescence excitation is in the vertical direction. The *G* is expressed as a corrected factor.

### 3.9. Effect of SA on S. aureus Membrane Protein

The effect of SA on membrane protein of *S. aureus* was determined by the reported method with minor modifications [27]. Logarithmic phase *S. aureus* cells were washed twice and re-suspended to make the concentration of 1 × 10^10^ CFU/mL in 0.85% saline solution. An amount of 0.3 mL of bacterial suspensions was added to 2.7 mL of KI or SA solution with different concentrations. After incubation for 1 h at room temperature, the fluorescence was measured using a fluorescence spectrophotometer (G9800A, Agilent). The emission spectra of this experiment were scanned from 280 to 400 nm, and the excitation wavelength was set at 258 or 296 nm.

### 3.10. Statistical Analysis

All experiments in this study were performed in triplicate. Statistical analyses were carried out by the SPSS software (version 20.0; SPSS, I., Chicago, IL, USA). The data were expressed as the mean values ± standard deviation (*n* = 3). A probability value at *p* < 0.05 was considered statistically significant.

## 4. Conclusions

The antibacterial mechanism of SA to *S. aureus* was investigated by examining membrane permeability, membrane potential, membrane integrity, TEM, membrane fluidity and membrane protein binding assays. Based on the data obtained, it could be concluded that the antimicrobial effect of SA against *S. aureus* is attributed to its action of SA to *S. aureus* cell membrane, which consequently increases *S. aureus* cell membrane permeability and destroys the membrane integrity, eventually inducing growth inhibition and bacterial death. A significant change of membrane fluidity of *S. aureus* treated with SA was observed, indicating the action of SA to *S. aureus* is due to the interaction of SA with the bacteria membrane lipids. Results further demonstrated that the antibacterial activity of SA against *S. aureus* could be caused by the interactions of SA with membrane proteins. In general, the bactericidal mechanism involves damaging the cell membrane and other cellular components. Our study provides a novel insight for studying the antimicrobial action of SA to *S. aureus* and using SA as a potential alternative food preservative in food industry.
